# Purulent Arthritis Treated by Simple Arthrotomy and Immediate Active Mobilization

**Published:** 1918-12

**Authors:** 


**Purulent Arthritis Treated by Simple Arthrotomy and Immediate Active Mobilization.** By CH. WILLEMS. *Bulletin de la Société de Chirurgie de Paris.* June 25, 1918.

The author states having obtained most excellent results in the treatment of purulent arthritis simply by means of arthrotomy and active mobilization of the injured joint.

Such a treatment is given in order to realize a thorough drainage of the wound. As yet no method has proved truly efficient for the drainage of injured joints: neither incisions nor swabbings of any kind. Hence arthrotomy has seemed a failure, and resection the only available method in cases of purulent arthritis.

Now active mobilization is recognized to permit of complete drainage and it will no longer be necessary to resort to resection.

*Mode of Action. Knee.* A vertical linear incision having been practised, to every muscular contraction of the joint corresponds the outflowing of a certain amount of pus; and if the movements are sufficiently frequent and extend far enough, no retention of pus need to be feared.

Hence the three following conditions ought to be realized: the incision should extend far enough above and beneath the wound; the wound must constantly be kept from closing spontaneously, as long as drainage proves necessary; mobilization must be as effective as possible.

Generally two incisions, one on each side of the knee, should be practised; if one only proves sufficient it is recommended to incise the external side.

*Elbow.* Unilateral and external incision generally proves sufficient.

*Shoulder.* Anterior incision; if it is insufficient, a second incision at the posterior end of the deltoid should be practised.

*Tibio-Tarsal Joint.* External incision practised along the edge of the fibula and bent in front under the malleolus.

Arthrotomy of the *wrist* proves difficult because of all the tendons surrounding that joint.

*Healing of the Wound.* The wound heals just as an ordinary abcess would. Infection seems always strictly limited to the synovial membrane. The cartilaginous surfaces are not infected and keep in normal conditions.

After some weeks, at least, suppuration gradually stops, and at the same time the incisions are allowed to close progressively.

The author considers it advisable to suture partially the wound towards the end of the treatment, leaving just a narrow channel for the flowing out of the remaining pus. This should prevent all stiffening of the joint.

The consequences of drainages obtained by mobilization are most important. Thanks to that method, infection is localized in the synovial membrane, and abscesses around the joint are never dealt with. The temperature rarely rises above 38° C. in the evenings. If there is a sudden rise of temperature this is a certain proof of some retention of pus, either because mobilization is not effective enough, or because the incisions are too short.

The general condition of the patient remains excellent.

Furthermore this method is responsible for the maintenance of all movements of the joint; muscular atrophy does not interfere, and after recovery the joint proves absolutely normal. Ankylosis can always be avoided, however severe should arthritis prove.

*Technic of Mobilization.* It must be resorted to *immediately* after the arthrotomy is practised; it must be done by the patient himself, by contracting his own muscles; passive contractions have proved quite inefficient. The movements must be as continuous and extend as far as possible.

Patients suffering from purulent arthritis of the knee or tibiotarsal joint are allowed to walk about long before their wounds are closed.

The movements do not prove very painful; they are of course somewhat difficult to realize at first, but gradually and rapidly they can be carried out without any strain.

The patient recognizes very soon that painful sensations in his wound are connected with insufficient drainage and that the only way of getting rid of all pains is to perform most carefully the mobilization of the joint.

After the wound has been closed for some time, it might happen that painful swelling of the joint would take place; this sometimes follows some slight trauma but not necessarily. A puncture must be practised, after which all disturbances disappear.

Active mobilization is of course impossible either when the muscular ligaments of the joint are completely torn away, or when the bones are severely fractured.

*Results.* 20 purulent arthritis have been treated in the above described way : knee, 11 times; elbow, 4 times; tibio-tarsal joint 5 times.

13 perfect healings have been obtained; 2 healings with incomplete movements; 4 ankylosis; in one case resection had to be resorted to.

In order to have true statistics the five cases followed by ankylosis and resection should be put aside, as in all of them the joint could not be actively mobilized, owing to great losses of muscles and tendons.

Just as good results were obtained with unmanageable patients who did not care to do as they were advised.

Among the 13 perfect healings, 8 had presented severe bone fractures together with purulent arthritis.

